# Health Tract, No. 282

**Published:** 1868-03

**Authors:** 


HEALTH TRACT, No. 282.

### LIFE LENGTHENED.

1. Cultivate an equable temper; many a man has fallen dead, in a fit of passion.

2. Eat regularly, not over thrice a day, and nothing between meals.

3. Go to bed at regular hours. Get up as soon as you wake of your self and do not sleep in the daytime, at least not longer than ten minutes before noon.

4. Work always by the day, and not by the job.

5. Stop working before you are very much tired,—before you are "fagged out."

6. Cultivate a generous and an accommodating temper.

7. Never cross a bridge before you come to it; this will save half the troubles of life.

8. Never eat when you are not hungry, nor drink when you are not thirsty.

9. Let your appetite always come uninvited.

10. Cool off in a place greatly warmer than the one in which you have been exercising: this simple rule would prevent incalculable sickness and save millions of lives every year.

11. Never resist a call of nature for a single moment.

12. Never allow yourself to be chilled "through and through," it is this which destroys so many every year, in a few day's sickness, from Pneumonia, called by some lung fever or inflammation of the lungs.

13. Whoever drinks no liquids at meals will add years of pleasurable existence to his life. Of cold or warm drinks, the former are most pernicious; drinking at meals induces persons to eat more than they otherwise would, as any one can verify by experiment, and it is excess in eating which devastates the land with sickness, suffering and death.

14. After fifty years of age, if not a day laborer, and sedentary persons after forty, should eat but twice a day, in the morning and about four in the afternoon; persons can soon accustom themselves to a seven-hour interval between eating, thus giving the stomach rest; for every organ without adequate rest will "give out" prematurely.

15. Begin early to live under the benign influences of the christian religion, for it "has the promise of the life that now is, and of that which is to come."

